# Characteristics of Carbon-Fiber-Reinforced Polymer Face Sheet and Glass-Fiber-Reinforced Rigid Polyurethane Foam Sandwich Structures under Flexural and Compression Tests

**DOI:** 10.3390/ma16145101

**Published:** 2023-07-20

**Authors:** Harri Junaedi, Tabrej Khan, Tamer A. Sebaey

**Affiliations:** 1 Engineering Management Department, College of Engineering, Prince Sultan University, Riyadh 11586, Saudi Arabia; tkhan@psu.edu.sa (T.K.); tsebaey@psu.edu.sa (T.A.S.); 2 Mechanical Design and Production Department, Faculty of Engineering, Zagazig University, Zagazig 44519, Egypt

**Keywords:** sandwich structure, CFRP, polyurethane foam, glass fiber, mechanical properties, flexural, compression

## Abstract

Composite sandwich structures are extensively used in aircraft applications. Aircraft components are required to be robust and lightweight. Sandwich structures made of carbon-fiber-reinforced polymer as the facing sheets and milled-glass-fiber-reinforced rigid polyurethane foam with a different glass fiber content as the core structure were prepared. The influence of glass fiber content in the foam on the sandwich structure’s mechanical properties was investigated. Flexural and compression tests were performed to assess the mechanical properties of the sandwich structures. Visual inspection and an optical microscope were used to observe the morphology of the polyurethane composite foams at different contents. From the flexural test, the force, facing stress and core shear stress improved with the increase in the milled fiber loading with the maximum increase at 10 wt.% loading and then a drop. Meanwhile, the compression modulus and strength increased up to 20 wt.% loadings and then dropped subsequently. The increase in the polyurethane composite foam’s compression strength shifted the bending load’s failure type from facing crack failure into core shear failure. The loadings range of 8–10 wt.% showed a transitional of the bending loading failure type. The density of the foams increased with the increase in milled glass fiber loading. At 10 wt.% loading, the density increased by 20%, and it increased by 47% at 30 wt.% loading. At 30 wt% loading, the optical microscope images of the foam showed wall thinning and broken walls that were responsible for the drop in the mechanical properties of the sandwich.

## 1. Introduction

A composite sandwich structure consists of two composite face sheets and a core [1]. The facing skins are made of high-strength and rigid material, while the core is usually made of lightweight and porous material. The employment of sandwich structures as aircraft components offers the benefit of being lightweight while also providing a range of other beneficial mechanical, chemical and physical properties. Some of these properties include flexural strength, rigidity, impact toughness, compression strength, shear strength, durability and sound and thermal insulation [2].

The behavior of a sandwich structure is affected by facing skin properties, core shear strength properties, interfacial bonding between the facing skin and core and the thickness of the skin and core [3]. In a composite sandwich, most of the bending loads are carried by the facing skins of the composite. Meanwhile, the sandwich core mainly handles the transverse shear and normal loads [4]. Thus, the core shear strength is an essential factor when developing sandwich structures. The core is also the one that keeps the skins apart from each other to form a lightweight sandwich structure. The types of the core are usually made of porous or hollow materials such as honeycomb, corrugated, foam and lattice structures, which can be made of different lightweight materials such as aluminum and different types of polymers [4,5,6,7,8,9]. Different types of foam have been used as a core material of composite sandwiches, such as polyurethane foam (PUF) [10,11,12,13], aluminum foam [14,15], epoxy foam [16,17], phenolic foam [18], polyethylene foam [19], polypropylene foam [20] and many others. Foam consists of hollow or porous particles and the polymer as the matrix is called syntactic foam [21,22,23,24]. The hybridization of different types of the core, such as a PUF-filled lattice structure core, has also been studied [10].

Meanwhile, for facing skins, fiber-reinforced polymers (FRPs), such as glass-fiber-reinforced polymer (GFRP), carbon-fiber-reinforced polymer (CFRP) and aluminum alloy, have become an option due to their excellent properties in terms of strength, stiffness and light weight. Interfacial bonding between the facing skins and core is also critical [25]. Without good interfacial adhesion, the load transfer mechanism from the facing skin to the core will not occur [26]. The sandwich would eventually fail in the interfacial due to transverse shear force at a lower force. An adhesive is usually added in the interfacial to promote bonding [27]. For the foam type of core, the direct casting of the foam between the facing sheets can provide a good interfacial adhesion between the core and the facing sheets without an additional adhesive.

PUF, as a core material, has lightweight, low-density and good thermal and sound insulation properties. In general, PUF and other foam materials have an advantage compared to other core materials in the ease of shaping and forming. Adding fillers to foam materials has been proven as a benefit to enhance the properties and expand the applications of polyurethane foams. Polyurethane foams’ compression modulus and strength can be improved by adding glass fiber (GF) [28] or carbon fiber (CF) [29]. Adding filler will also alter the cell morphology of the PUF cell [30,31]. Cell morphology affects the foam’s mechanical and physical properties [32].

Several investigations on filler-reinforced foam core sandwich structures have been conducted. Mahfuz et al. [33] studied the effect of TiO_2_ nanoparticle loading into a PUF core on a sandwich panel’s flexural and fracture toughness properties. The skins were made of a glass-fiber-reinforced epoxy laminate. They reported a significant enhancement in the flexural strength and modulus at the 3 wt.% loading of TiO_2_ nanoparticles. However, adding the nanoparticles reduced the debond fracture toughness of the face sheet and the core. Caglayan et al. [34] investigated the effects of a carbon nanotube (CNT) on a sandwich panel’s rigid polyurethane (PU) core. Four plies of prepreg carbon-fiber-reinforced epoxy were used as the facing skins of the sandwich panel. As a result, an increase of 13% in compressive strength compared to neat PUF was reported. Core shear and face ultimate strength also improved by 30%. The core shear failure type occurred for all sandwich panels. Devaraj et al. [25] embedded chopped fiber on the interface of the face skin and core of a sandwich panel. Three different types of chopped fibers with an average length of 12 mm were used: glass, carbon and Kevlar. The properties of the sandwich panels were investigated with flexural, compressive, shear and fracture toughness tests. The sandwich panels with chopped glass fibers presented the highest face–core interfacial bonding, flexural strength, shear strength and fracture toughness compared to other chopped fibers added to the interfacial region. To the best of the authors’ knowledge, the data available on a polymeric sandwich with a milled-fiber-reinforced PUF core are limited, which this study addresses.

The current paper studied the effects of a different GF content in the PUF of a CFRP-GF-reinforced PUF sandwich composite. Different wt.% of GFs were added to PUF to form polyurethane composite foam. The polyurethane composite foams were used in the sandwich structure as a core, with CFRP as the facing skin. Flexural and compression tests were carried out to evaluate the composite sandwiches’ mechanical properties. Flexural strength, core shear strength and facing strength were determined from the flexural test. Meanwhile, the compression strength and modulus of the PUF core were calculated with a compression test. An optical microscope was used to observe the transformation in the cell morphology of the foam due to the addition of GF.

## 2. Materials and Methods

### 2.1. Materials

An as-received composite laminate (CFRP) of a 0.85 mm thickness was supplied by Dragonplate, ALLRed & Associates Inc. (Elbridge, NY, USA). It was employed as the facing sheet of the sandwich. The CFRP laminate was quasi-isotropic and consisted of three plies of carbon fiber (CF) with twill woven fabric at a 0/90° orientation on the front outer ply, spread tow twill woven at a ±45° orientation on the middle ply and plain twill woven at a 0/90° orientation on the back outer ply with a total thickness of 0.85 mm and epoxy 304 as the matrix. The density of the CFRP was 1.42 g/cm^3^. Based on the ignition loss method according to ASTM D2584-18, the fiber volume fraction of the CFRP was 52%. The closed-cell rigid PUF components, from a manufacturer, with a cured density of about 96 kg/m^3^ (6 lb/ft^3^) were supplied from Totalboat (Bristol, RI, USA). The PUF was used as the core material of the composite sandwich. The PUF comprised two components (components A and B) with a mix ratio of 1:1 by weight. Component A contained polymethylene polyphenyl polyisocyanate, and component B consisted of diethylene glycol and 1,1,1,3,3-Pentafluoropropane (HFC-245FA) as a blowing agent. Additionally, 1/32 inch mesh milled glass fiber with a diameter of 16 μm and an average length of 230 μm [35] was used as reinforcement. The sizing was a cationic surface sizing agent. The glass fiber was supplied by Fibre Glast Developments Corp (Brookville, OH, USA).

**Figure 1:**
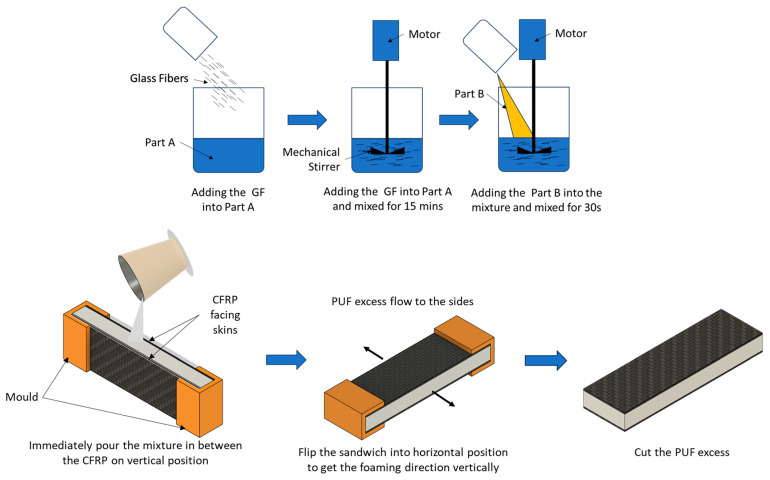
Manufacturing process of the sandwich composite.

The CFRP composite laminates were cut with a band saw into a dimension of 200 × 30 mm. The core thickness was set to 10 mm to achieve a total thickness of 11.7 mm. The core structure was a glass-fiber-reinforced PUF with different-content glass fiber, i.e., 0, 2, 4, 6, 8, 10, 20 and 30 wt.%. For the polyurethane composite foam, first, component A of the PUF was mixed with the GF at the prescribed content using a mechanical stirrer for 15 min. Then, component B was added to the mixture and stirred for about 30 s. The mixture was immediately poured between 2 CFRP sheets, keeping the distance between the 2 sheets at 10 mm. The joint between the skins and the core was due to directly casting the PUF in between the skins (Figure 1). During the foaming process, the face sheets were set horizontally to obtain the foaming direction in the vertical direction, which is the loading direction. To ensure a uniform thickness of the sandwich, the two face sheets were set in a die of the required thickness during the foaming process. The excess foam was left to expand outside the sandwich and cut after the foam had fully cured.

### 2.2. Methods

**Figure 2:**
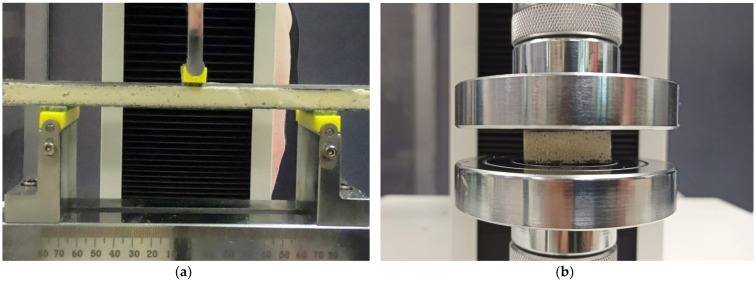
Mechanical testing setup for (**a**) flexural test and (**b**) compression test.

Flexural and compression tests were performed using a universal testing machine (WDW-10, Dongguan Hongtuo Instruments Co., Ltd., Dongguan, China). The three-point flexural test was performed with a crosshead speed of 2 mm/min until failure for a batch of three samples for each configuration according to the ASTM D7249 standard [36]. Facing stress (σ) and core shear stress ($F_{s}^{ult}$) at maximum forces are calculated by Equations (1) and (2), respectively, as follows:

(1) $$\sigma =\frac{P_{max}}{\left(d+c\right)b}$$

(2) $$F_{s}^{ult}=\frac{PS}{2t(d+c)b}$$

where *P_max_* is the maximum force from the force–deflection curve, *b* is the width of the sandwich, *c* is the core thickness, *d* is the total thickness of the sandwich, *S* is the support span of the bending test and *t* is the thickness of the facing skin. The span support (*S*) of the specimen was set to 150 mm. The specimen had a nominal length of 200 mm, a width of 30 mm and a thickness of 11.7 mm. During loading, the sample edge was monitored to examine any damage on the foam surface and/or the separation of the face sheets from the core. Figure 2a shows the three-point bending test of the sandwich panel. Meanwhile, a compression test was done to assess the compression properties of the core material. The test was performed on the composite sandwiches according to the ASTM C365 standard [37] with a 0.5 mm/min crosshead speed, as shown in Figure 2b. A specimen of 30 × 30 mm and a foam thickness of 10 mm was used. The specimens were compressed up to 90% of strain. The compression strength and modulus of the PU composite foam core were calculated. Five compression tests were performed for each composition.

An optical microscope was utilized to observe the cell morphology of the PUF and the alteration of the cell morphology due to the addition of milled GF. The apparent density of the PU foam and PU composite foams were measured by dividing the weight by the foam volume to identify the change in the density due to the addition of GF.

## 3. Results and Discussion

Table 1 lists the apparent density of the composite foam core. As anticipated, the density of the PU composite foam increased with the increase in the wt.% of GF loading since the GF has a much higher density (2.54 g/cm^3^) compared to the neat PUF. At 30 wt.% of GF loading, the density increase was 46% compared to the neat PUF. The same phenomenon has also been reported by other researchers [38,39].

**Figure 3:**
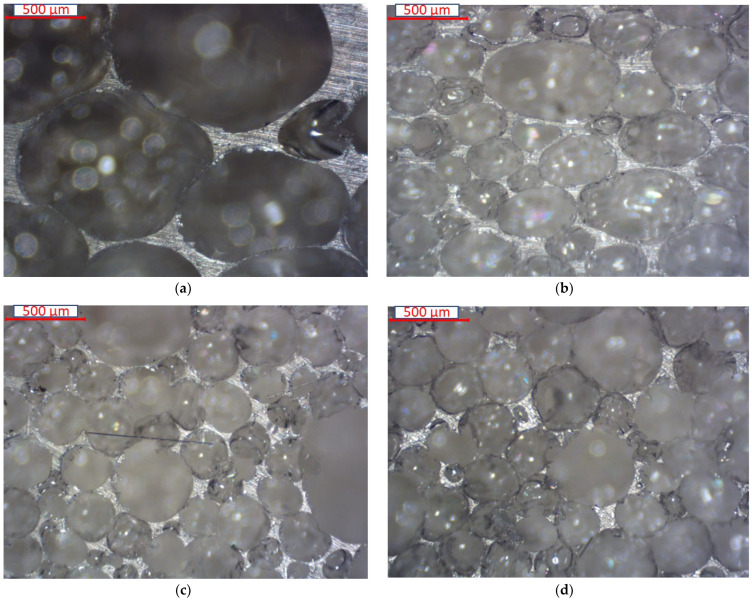
Optical microscope (OM) images of the foam core: (**a**) pure PUF, (**b**) PUF/10% GF, (**c**) 20% GF and (**d**) PUF/30% GF.

**Figure 4:**
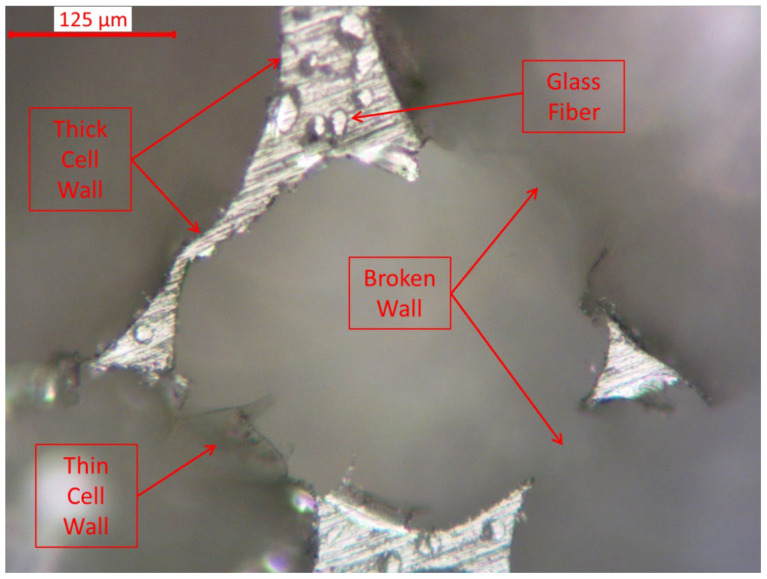
Optical microscope (OM) images of the foam core of PUF/30% GF at a higher magnification.

Figure 3 shows the optical microscope images of PU composite foam at different wt% GF loading. It shows that the addition of GF reduces the cell size and wall thickness of the foam. At 30 wt% of GF, it can be observed that the wall thickness is getting thinner compared to lower GF loadings. Our findings are in agreement with others [38,40]. For a deeper investigation, Figure 4 shows the cell of the foam of PUF/30% GF at a higher magnification. From the figure, it is obvious that fibers tend to embed in the wall of the cell. In each cell, thick, thin and broken walls can be found. Thin and broken walls are responsible for the deterioration of the strength of the foam.

**Figure 5:**
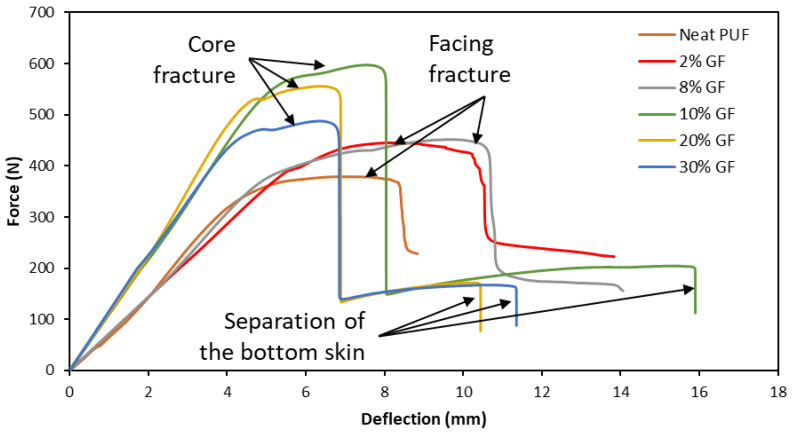
Representation of the flexural-force–deflection curves for different configurations.

Figure 5 shows the flexural-force–deflection curves of the composite sandwiches with different GF contents. There are two types of curves observed. For the first one, once reaching the maximum load, a sudden drop occurs until a particular load, then continues to drop slowly. This behavior is associated with the failure of the face sheet at the contact point with the test fixture. The second form of the load–displacement curve has two steps of failure; after reaching the maximum load, the load dropped suddenly due to the core shear failure, then the load started to increase again, followed by a second sudden drop due to the separation of the bottom skin. For 8 wt.% GF and below, damage associated with the face sheet was observed. For 10 wt.% GF and above, the second damage mechanism was observed.

**Figure 6:**
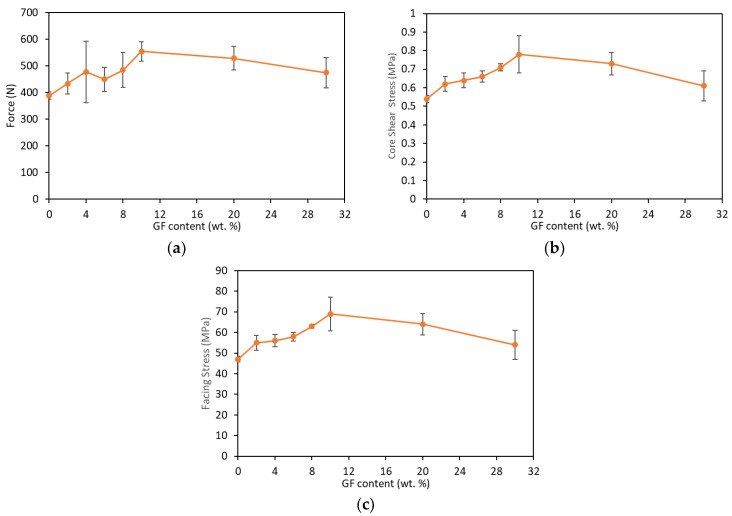
Flexural properties of the composite sandwich: (**a**) Force, (**b**) Core shear stress and (**c**) Facing stress (at different wt.% of GF).

From the force–deflection curves, core ultimate shear strength and facing strength can be calculated based on the ASTM D7249 standard [36]. Figure 6 shows graphs of force, core shear ultimate strength and facing strength by increasing GF content. The force, core shear stress and facing stress have a similar tendency: to increase up to 10 wt.% loadings and subsequently drop. The sandwich of 10 wt.% of GF improved the force, core shear strength and facing strength by 43%, 39% and 40%, respectively, compared to the sandwich with neat PUF. The presence of GF improved the flexural strength of the sandwiches. The embedded GF on the PUF wall and the decrease in the cell size contribute to the improvement in the flexural properties. Meanwhile, the decrease in the force, core shear stress and facing stress of the sandwiches at 20 wt.% of loading and above was due to the agglomeration of GF. An excessive loading of GF on the foam disrupts the cell structure, reduces the wall thickness and furthermore even breaks the wall, thus creating a stress concentration in the foam wall and decreasing the flexibility of the foam [41]. It is worth remarking that the facing stress, although it is different in value, reaches its maximum at almost the same deformation level (around a 8 mm deflection). After this level, degradation starts with a high rate in the neat PUF and is slower as the wt.% of GF increases up to 10 wt.%, where a sudden fracture occurs. 

**Figure 7:**
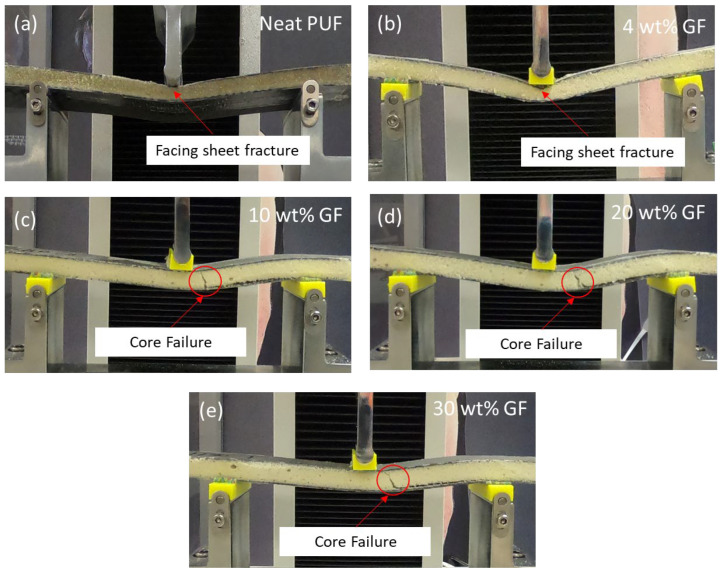
Sample failure types for sandwich composite: (**a**) neat PUF, (**b**) 4 wt.% GF, (**c**) 10 wt.% GF, (**d**) 20 wt.% GF and (**e**) 30 wt.% GF.

The damage associated with each type of failure is shown in Figure 7. Two types of failure were observed from the failed samples: top-facing skin failure and core shear failure. The type of failure was affected by the wt.% loading of the GF. No failure begins from interfacial bonding between the facing and the core foam, which confirms the hypotheses of high interface bonding between the PUF and the CFRP face sheet. Top skin failure occurred due to a compression load on the foam, creating local bending and fiber kinking on the top-facing skin, then leading to a fracture of the top-facing skin. The core failure occurred due to the shear stress on the foam exceeding the shear strength of the foam. Core failure was then followed by the separation of the bottom-facing skin. In the case of separation, a thin layer of foam was observed as attached to the surface of CFRP. This indicates higher interfacial bonding between the facing skin CFRP and the PU composite foam core, as compared to the shear strength of the core [21].

**Figure 8:**
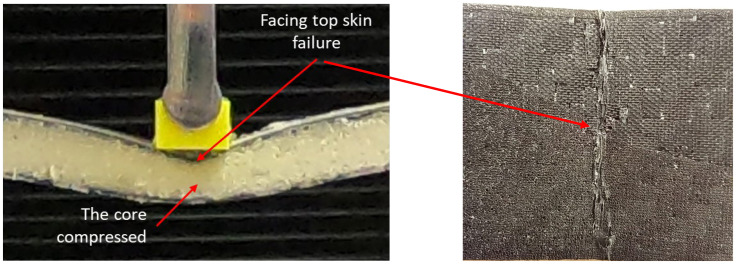
Close-up image of flexural test sample failure of 4 wt.% GF.

As seen in Figure 8, a close-up image of the 4 wt.% GF sample, the core is compressed due to the bending load, thus causing the top-facing skin failure due to local bending. The top skin experienced a higher deflection compared to the bottom-facing skin. The same phenomenon has also been reported by Steeves and Fleck [42] for FRP-facing and foam core composite sandwiches. It was described as a failure due to indentation.

**Figure 9:**
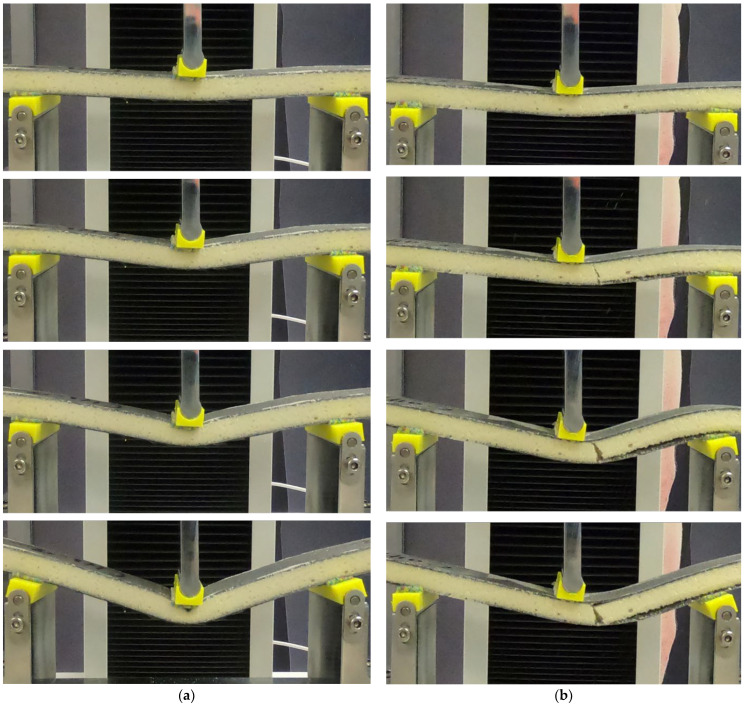
The flexural test failure mechanism: (**a**) 8 wt.% GF with top-facing skin failure and (**b**) 10 wt.% GF with core shear failure.

A failure mechanism comparison of the composite sandwich with facing failure at 8 wt.% and core failure at 10 wt.% is shown in Figure 9. For the facing failure sample, Figure 9a shows that the failure starts from the crushing of the foam core and is followed by the break of the top-facing skin material. Further bending to the sample compressed more of the core foam. Meanwhile, for the core failure sample (Figure 9b), the failure started from the fracture of the core due to transverse shear stress, which was then followed by a partial separation of the bottom-facing skin. With the increase in the deflection, the bottom-facing skin was opened, followed by a full separation of the right side of the load pin on the bottom-facing skin and partial top-facing skin. From Figure 5, it can be observed that deflection increases with the increase in GF for GF lower than 8 wt.%. The increase in the deflection was related to the improvement in the compression strength composite foam. The localized bending on the top of CFRP facing due to localized indentation was detained as the compression strength of the PU composite foam increased. As a result, the facing failure occurred at a higher deflection.

**Figure 10:**
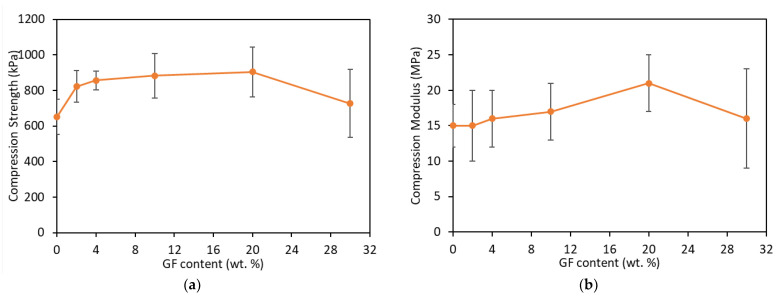
Compression properties of the composite sandwiches: (**a**) strength and (**b**) modulus at different wt.% of GF.

To thoroughly understand the behavior of the sandwiches under flexural loading with a different wt.% of GF, the compression properties need to be investigated as correlated to the damage mechanism in bending. The compression strength was calculated with the stress at 10% of strain divided by the sample’s cross-section area. The compression modulus was evaluated from the slope of the linear section of the compression stress–strain curve. Figure 10 shows the compression properties of the sandwiches by the increase in wt.% of GF. An enhancement in compression strength was observed, as seen in Figure 10a. An increase of about 26% compared to PUF only occurred with the addition of 2% of GF, but then the increase was not as much as before with the increase in wt.% of GF. The maximum increase was observed at 20 wt.% GF, with about a 38% increase compared to PUF only before it dropped at 30 wt.% of GF loading. Other researchers [39,43] also reported an increase in the compression strength of PUF due to reinforcement by GF. There was a slight increase in the compression modulus up to 10 wt.% GF loading. A 40% improvement was observed at 20 wt.% GF loading followed by a drop at 30 wt.%. 

Adding GF into PUF enhances the compression strength and modulus, nevertheless decreasing the flexibility of the PU composite foam. The increase in the strength and modulus of the core hindered the premature crushing of the PU composite core under the loading pin during the bending. Facing skin failure that occurred for sandwiches with neat PUF and PU composite foam with lower GF loadings did not arise in sandwiches at a GF loading of 20 wt.% or higher. The sandwiches with a higher wt.% of GF tend to fail with a core shear fracture. The decrease in the compression strength of the core due to the excessive amount of GF disturbed the foaming and continuity of the foam’s cell structure [44].

## 4. Conclusions

Composite sandwich panels consisting of CFRP as facing skins and GF-reinforced PUF as the core were manufactured. Adding GF into polyurethane foam has a constructive effect on the mechanical and physical properties of the sandwich composite. The density of the PU composite foam increased with the increase in GF loading. The force, core shear stress and facing stress of the sandwich composite was enhanced up to about 40% at 10 wt.% GF loading and then dropped subsequently. The compression strength and modulus also improved with increased GF loading, with the maximum improvement at 20 wt.% GF loading and then a drop. The failure of the sample on the flexural test shifted from the top-facing skin crack into core shear failure in the range of 8–10 wt.% GF loadings. Adding GF to the PUF increases the compression strength of the PU composite foam, thus preventing the crushing or indentation of the core. But the higher content of the GF also reduced the wall thickness and created the agglomeration of GF on the wall, which reduced the flexibility of the core foam; therefore, the sandwich failed at a lower force.

## Figures and Tables

**Table 1:** The apparent density of polyurethane composite foam.

wt.% GF	PUF Apparent Density (kg/m^3^)
0	90
4	96
10	108
20	113
30	132

## Data Availability

The data will be made available upon request to the authors.
